# Metastasis of a Malignant Thymoma Presenting as a Right Atrial Mass

**Published:** 2020-01

**Authors:** Timoor Etemad, Narges Shahbazi, Ali Hosseinsabet, Khalil Forozannia

**Affiliations:** 1 Department of Cardiology, Tehran Heart Center, Tehran University of Medical Sciences, North Karegar Street, Tehran, Iran. 1411713138. Tel: +98 21 88029731. Fax: +98 21 88029731. E-mail: dr_taimoor_etemad@yahoo.com.; 2 Assistant Professor of Pathology, Department of Pathology, Tehran Heart Center, Tehran University of Medical Sciences, North Karegar Street, Tehran, Iran. 1411713138. Tel: +982188029233. Fax: +982166175431. E-mail: nshahbazi@sina.tums.ac.ir.; 3 Associate Professor of Cardiology, Department of Cardiology, Tehran Heart Center, Tehran University of Medical Sciences, North Karegar Street, Tehran, Iran. 1411713138. Tel: +98 21 88029731. Fax: +98 21 88029731. E-mail: ali_hosseinsabet@yahoo.com.; 4 Professor of Cardiac Surgery, Department of Cardiovascular Surgery, Tehran Heart Center, Tehran University of Medical Sciences, North Karegar Street, Tehran, Iran. 1411713138. Tel: +98 21 88029731. Fax: +98 21 88029731. E-mail: drforouzan_nia@yahoo.com.

Dear editor,

A 44-year-old man referred to our hospital because of a right atrial (RA) mass. The patient had a 1-year history of a malignant thymoma, for which he had undergone radiotherapy and chemotherapy. Initially, he was admitted into another hospital due to fever and dyspnea and his blood culture revealed *Alcaligenes sp*. His response to antibiotic therapy was poor; consequently, he underwent transthoracic and transesophageal echocardiographic examinations, which demonstrated a nonhomogeneous fixed mass attached to the roof of the RA. Additionally, there were some mobile particles on the RA mass and the superior vena cava catheter (implanted for chemotherapy) was free of any particles. His attending physician decided to remove the catheter as a possible source of infection, which was accompanied by clinical response to antibiotic therapy. 

For further treatment, the patient was referred to our hospital, which is a tertiary cardiology and cardiovascular surgery center. Repeated transthoracic echocardiography confirmed the presence of the aforementioned RA mass (Figure 1, Video 1), and the surgical removal of this mass was planned. In the operating room, a mediastinal mass and the RA mass were removed. The pathological examination of these masses revealed characteristic epithelial tumor lobules distributed in a fibrotic tissue. The intracardiac mass showed sheets and lobules of the same highly atypical epithelial cells infiltrated by some lymphocytes. (Figure 2). Postoperative transthoracic echocardiography before discharge demonstrated no residual tumor in the cardiac chambers. 

 In this case, infectious endocarditis, thrombosis, and tumor metastasis were at the top of the differential diagnosis list; nonetheless, tumor metastasis was finally documented. Thymoma metastasis has been reported from many organs such as the liver, lung, and bone.^1^^-^^3^ In regard to the heart, the metastatic involvement of the pericardium, the superior vena cava, and the RA by malignant thymomas has been reported.^4^^, ^^5^ Accordingly, in the evaluation of patients with a history of malignant thymomas and cardiac masses, tumor metastasis should be considered in the differential diagnosis. 

**Figure 1:**
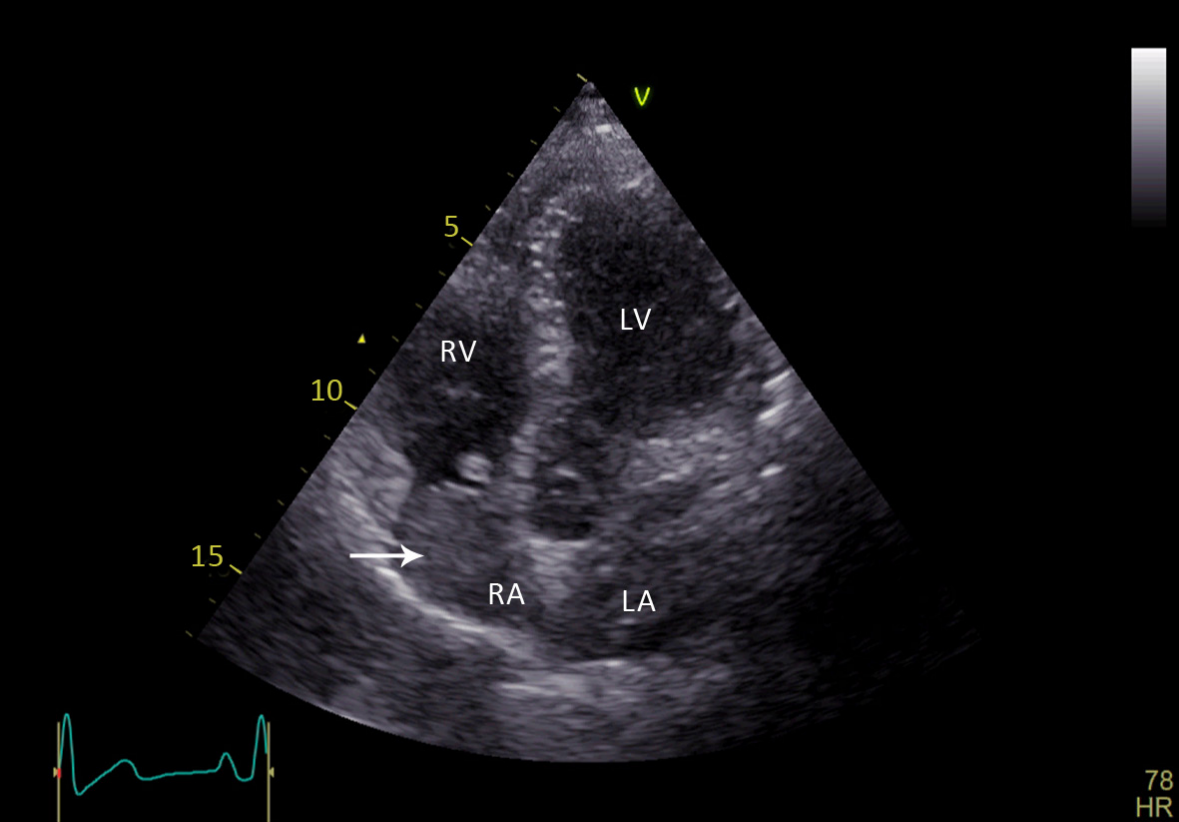
Transthoracic echocardiography (apical 4-chamber view) shows a fixed right atrial nonhomogeneous mass, which is attached to the roof of the right atrium.

LA, Left atrium; LV, Left ventricle; RA, Right atrium; RV: Right ventricle

**Figure 2:**
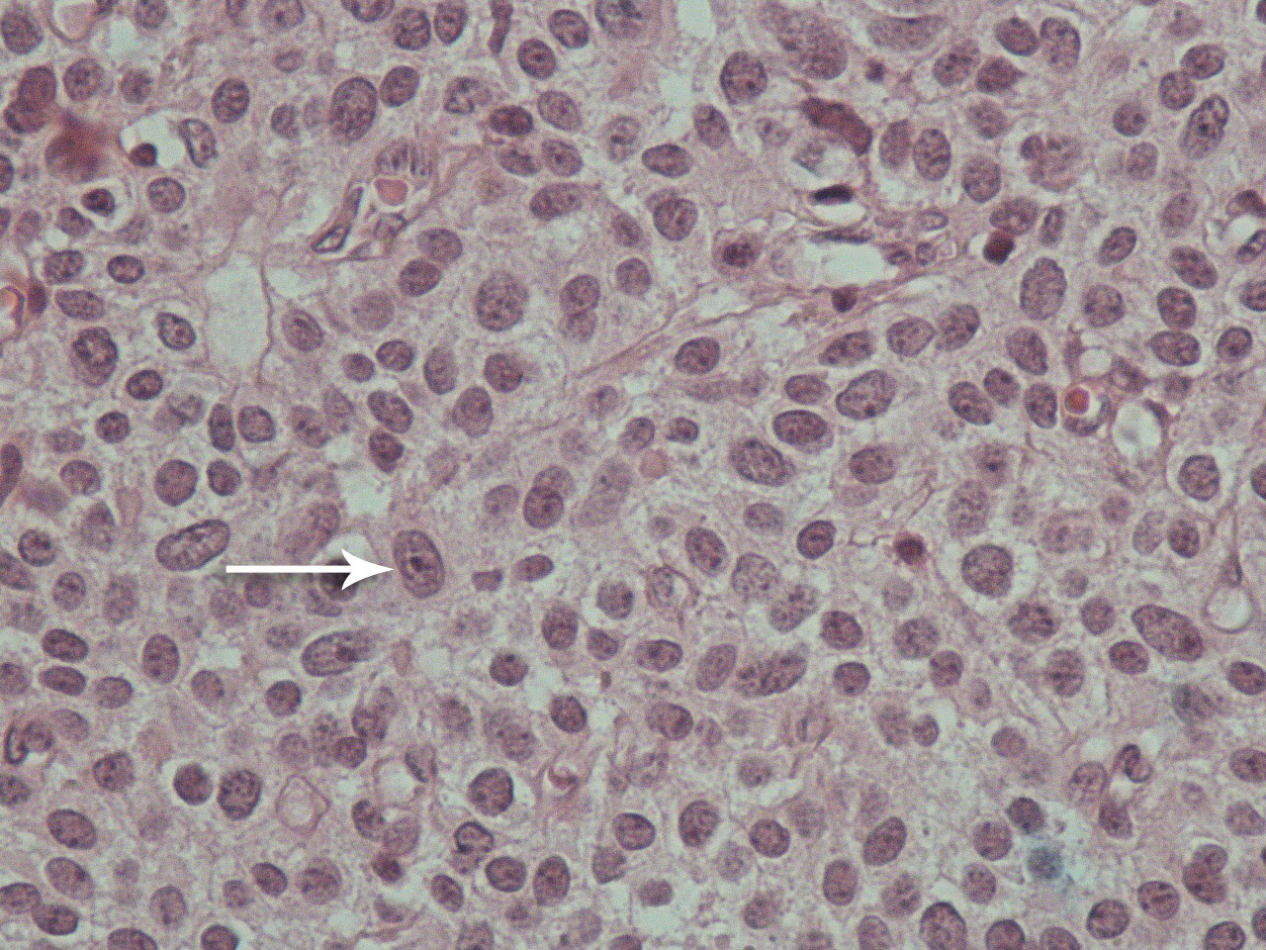
Microscopic evaluation of the surgically removed mass shows atypical epithelial cells (arrow) in sheets in an intracardiac metastatic thymic carcinoma

## Notes:

***To watch the following videos, please refer to the relevant URLs:***

http://jthc.tums.ac.ir/index.php/jthc/article/view/1026/891

Video 1. Fixed right atrial nonhomogeneous mass is attached to the roof of the right atrium (apical 5-chamber view of transthoracic echocardiography).
